# Improving the differential diagnosis between myelodysplastic syndromes and reactive peripheral cytopenias by multiparametric flow cytometry: the role of B-cell precursors

**DOI:** 10.1186/s13000-015-0259-3

**Published:** 2015-04-29

**Authors:** Suiellen C Reis-Alves, Fabiola Traina, Konradin Metze, Irene Lorand-Metze

**Affiliations:** Hematology and Hemotherapy Center, University of Campinas, Carlos Chagas Street, 480, 13083-878 Campinas, São Paulo, Brazil; Faculty of Medicine of Ribeirão Preto, University of São Paulo, Vila Monte Alegre, 14048-900, Ribeirão Preto, Sao Paulo, Brazil; Faculty of Medicine, University of Campinas, Tessália Vieira de Camargo Street 126, 13083-887, Campinas, São Paulo, Brazil

**Keywords:** MDS, Flow cytometry, CD34^+^ cells, B-cell precursors, Diagnosis

## Abstract

**Background:**

Immunophenotyping is a valuable ancillary technique for the differential diagnosis between myelodysplastic syndromes (MDS) with low bone marrow (BM) blast counts and a normal karyotype, and reactive peripheral (PB) cytopenias. Our aim was to search for the most important variables for this purpose. We also analyzed the age variation of BM B-cell precursors (BCP) and its differences in reactive and clonal cytopenias.

**Methods:**

Immunophenotypic analyzes were performed in BM of 54 patients with MDS (76% with BM blasts <5%) and 35 cases of reactive cytopenias. Healthy allogeneic BM transplantation donors (n = 41) were used as controls. We used a four-color panel of antibodies analyzing 9 granulocytic, 8 monocytic and 6 CD34^+^ cell features.

**Results:**

Asynchronous shift to the left in maturing granulocytes and increase in CD16^+^ monocytes were also found in reactive PB cytopenias, but the most important aberrancies in MDS were seen in myeloid CD34^+^ cells. Decrease in BCP, that is a hallmark of MDS, could also be found in reactive cytopenias, especially in patients >55 years. % BM BCP could be calculated by the formula: *(−7.97 × log age) + (4.24 × log % CD34*^*+*^*cells) – (0.22 x nr. alterations CD34*^*+*^*cells) + 0.577.* Corrected R^2^ = 0.467.

**Conclusion:**

Analysis of myelomonocytic precursors and CD34^+^ cells was satisfactory for the differential diagnosis between reactive PB cytopenias and MDS. The most specific alterations were found in CD34^+^ cells. Comparison of the values obtained with those of normal age-matched controls is recommended.

## Background

In the last years, numerous studies have confirmed the utility of multiparametric flow cytometry (FCM) in the diagnosis of myelodysplastic syndromes, especially in cases with a normal karyotype, and its differential diagnosis with peripheral cytopenias of non-clonal origin [1-10]. FCM of BM hemopoietic precursors has been focused mainly on myelomonocytic precursors and CD34^+^ progenitors. There is no single specific abnormality, but the presence of three or more aberrancies may strongly support the diagnosis of MDS [1,2].

Several kinds of phenotypic abnormalities have been described in MDS such as a low SSC in granulocytic precursors, loss of antigen expression, asynchronous maturation or maturation block, aberrant cross-lineage co-expressions, quantitative and qualitative abnormalities of CD34^+^ cells, along with the decrease of precursor B cells (BCP) [9,11-21]. Many phenotypic abnormalities found in CD34^+^ cells have been associated with disease progression and are able to predict a shorter survival of the patients [5,8,10,15,17,19,20,22-31].

According to the European Leukemia Net Working Group (ELN) standardization [3,6,31], BM immunophenotyping in MDS should at least focus on the maturation of myelo-monocytic precursors as well as the enumeration of hemopoietic progenitors and BCP. So, a minimal panel should be designed to detect all these abnormalities [3,5,8,22,31]. Furthermore, comparison with the normal pattern of antigen expression of each lineage and maturation step should be performed. Besides, several scores based on phenotypic findings have been described to support the differential diagnosis between MDS and reactive PB cytopenias, but there is no general consensus indicating the best one for application in daily routine [5,8,10,15,17,23,25-31].

In our previous studies [10,19,23], we have analyzed the utility of a four-color panel that was able to detect several phenotypic abnormalities in the myelomonocytic series and CD34^+^ cells. We have also found that maturation abnormalities of myelomonocytic precursors are similarly present in all WHO types of MDS, while those detected in CD34^+^ cells are the most important to predict a shorter survival of the patients [19,20]. Recently, comparing the prognostic value of IPSS, IPSS-R and WPSS with those obtained by flow cytometry, we found that CD34^+^/CD13^+^ cells and total number of phenotypic alterations found in the myelomonocytic series and CD34^+^ cells were additional independent prognostic factors to the clinical scores [23].

Here, our aim was to examine which abnormalities detected by our panel were more important for the differential diagnosis between reactive PB cytopenias and cases of MDS with a normal karyotype. As the number of BM B-cell precursors is age-dependent, we also examined the impact of this variation in the utility of this feature in the differential diagnosis.

## Methods

### Patients and samples

Since December of 2009, immunophenotyping was included in the diagnostic work-up of peripheral cytopenias in our Institution together with PB counts, BM cytology and karyotyping. The WHO criteria were used for the diagnosis of MDS and exclusion of deficiency anemias, viral infections, auto-imune diseases and renal or hepatic insufficiency was made [1,2]. During the period of the study (December 2009 – February 2013), we could confirm the diagnosis of MDS in 56 cases while in 35 cases the final diagnosis of reactive cytopenias was made (Table 1). Twenty five patients were excluded because of lack of complete clinical data or uncertain diagnosis.

**Table 1:** **Clinical and hematological features of MDS patients**

**N° of patients**	**54**
**Sex (male/female)**	34/20
**Age, median years (range)**	69 (15–84)
**WHO classification, n° (%) of the patients**	
RA	7 (13)
RCMD	24 (45)
RCMD-RS	11 (20)
RAEB-1	6 (11)
RAEB-2	6 (11)
**IPSS-R risk group of the patients**	
Very Low	5
Low	17
Intermediate	16
High	10
Very High	3
**IPSS risk group of the patients**	
Low	15
Intermediate-1	25
Intermediate-2	9
High	2
**WPSS of the patients**	
Very low	3
Low	12
Intermediate	22
High	12
Very High	2

WHO, World Health Organization; RA, refractory anemia; RCMD, refractory cytopenia with multilineage dysplasia; RAEB, refractory anemia with excess blasts; IPSS International Prognostic Scoring System; WPSS, WHO classification- based prognostic scoring system; ANC, absolute neutrophil count.

The classification of the MDS cases was made according to the WHO criteria and risk category according to IPSS, IPSS-R [32] and WPSS (using hemoglobin values instead of “transfusion dependency”) [33] was assessed.

Cytogenetic analysis of BM was performed after 24 hours of culture according to standard methods. In each case, at least 20 mitoses were analyzed and the karyotypes were reported according to the International System for Human Cytogenetic Nomenclature [34].

Normal BM samples were obtained from 41 healthy donors for allogeneic bone marrow transplantation (age: 15–69 years) in order to standardize a normal immunophenotypic profile for our laboratory. All samples were collected during the period between July 2009 and January 2013.

All BM samples were obtained after informed consent was given by each person, according to the recommendations of the local Ethics Committee (proc. Nr. 0652.0.146.000-08).

### Flow cytometry analyses

Immunophenotyping was performed as previously described [10,19]. Briefly, the EDTA-anticoagulated BM sample (5-7 × 10^6^ cells in 100 μl per test) was processed using a standardized direct lyse-and-wash technique within 24 hours after bone marrow aspiration [3]. Quality control, calibration and compensation with FACS Comp were performed daily in our equipment.

Antigenic expression was studied using four-color combinations of monoclonal antibodies (MoAbs) conjugated with fluorescein isothiocyanate (FITC), phycoerythrin (PE), peridin clorophyll protein (PerCP) and allophyicocyanin (APC) fluorocromes. The following combinations were used to study the myelomonocytic maturation and progenitor cell populations: HLA-DR/CD14/CD45/CD33; CD16/CD11b/CD45/CD13; CD13/CD34/CD45/CD117; CD10/CD19/CD45/CD34; CD7/CD56/CD45/CD34. The specificity and source of each reagent have already been described in detail [23]. Immediately after staining, samples were acquired in a FACS Calibur flow cytometer (Becton Dickinson – BD Biosciences) using the CellQuest software (BD Biosciences). Instrument quality control, calibration using FacsComp™ (BD) software and spectral compensation were performed daily. Information about at least 100,000 nucleated BM cells was acquired for each sample. Data analysis was made using Infinicyt software (Cytognos). The strategies of analysis were standardized as previously described [10,19,23]. These variables also were assessed in normal BM.

The analysis of the myelomonocytic series was made as previously described [23] according to ELN standardization [3,27,31]. Briefly, maturation pattern of neutrophils was analyzed by their expression of CD13, CD16, CD11b, CD33 and HLA-DR. The side-scatter (SSC) of the granulocytic population and the antigen expressions were considered abnormal if the values of the mean fluorescence intensity (MFI) were above or below the benchmark values obtained for the normal cases (Table 2). Monocytes were analyzed by their expression of HLA-DR, CD64 and CD14. The combination CD16/CD11b/CD45/CD13 was used to quantify the CD16^+^ monocytes. The aberrant expression of CD34, CD7 and CD19 was investigated in each population considering abnormal when at least 10% of the cells expressed these antigens. For expression of CD56 in the myelomonocytic cell line, only values of >20% for granulocytes and >50% for monocytes were considered abnormal.

**Table 2:** **Comparison of flow cytometric features among normal, non-clonal cytopenias and MDS**

	**Normal values n = 41**	**Reactive cytopenias n = 35**	**MDS <5% BM blasts n = 42**	**MDS >5% BM blasts n = 12**	**p**
**Granulocytic series**					
SSC	521 (396–658)	534 (340–628)	394 (236–619)	462 (319–648)	<0.0001
CD45 MFI	138-577	265 (107–683)	216 (34–770)	270 (89–551)	0.02
Asynchronous shift to the left		7 (21%)	11 (26%)	6 (46%)	0.12
**Monocytic series**					
SSC	253 (196–399)	237 (149–390)	233 (66–399)	203 (184–322)	0.01
% monocytes	0.8-3.6	3.0 (0.7 - 8.8)	3.7 (0.2 - 18)	3.3 (0.1 - 21)	0.22
% CD16^+^ monocytes	<0.48	0.3 (0.1 - 3.4)	0.4 (0.02 - 10)	0.9 (0.05 - 4)	0.003
**Cells in the blast gate**					
BM % blasts (cytology)		1.0 (0.5 - 4.0)	1.0 (0.5 - 5)	12 (7.7 - 18)	
% CD34^+^ cells	<1.59	0.6 (0.02-2.1)	1.0 (0.02- 5.1)	3.6 (0.2 - 28)	<0.0001
% B cell precursors/total cells	>0.05 (0.04-0.53)	0.06 (0–0.7)	0.06 (0–0.7)	0.01 (0.01 - 0.14)	<0.0001
%CD34^+^/CD13^+^/CD117^+^	<0.62	0.3 (0.01-0.8)	0.5 (0.01-4.2)	1.4 (0–24.1)	0.001
% CD34^+^/CD7^+^	<0.08	0.05 (0–0.09)	0.04 (0–0.7)	0.13 (0.04-26)	<0.0001
% CD34^+^/CD56^+^	<0.05	0.01 (0–0.09)	0.03 (0–1.9)	0.13 (0.02-27)	<0.0001
**Total abnormalities**	**0 (0–2)**	**2 (0–5)**	**7 (3–13)**	**9 (6–16)**	<0.0001

**Figure 1:**
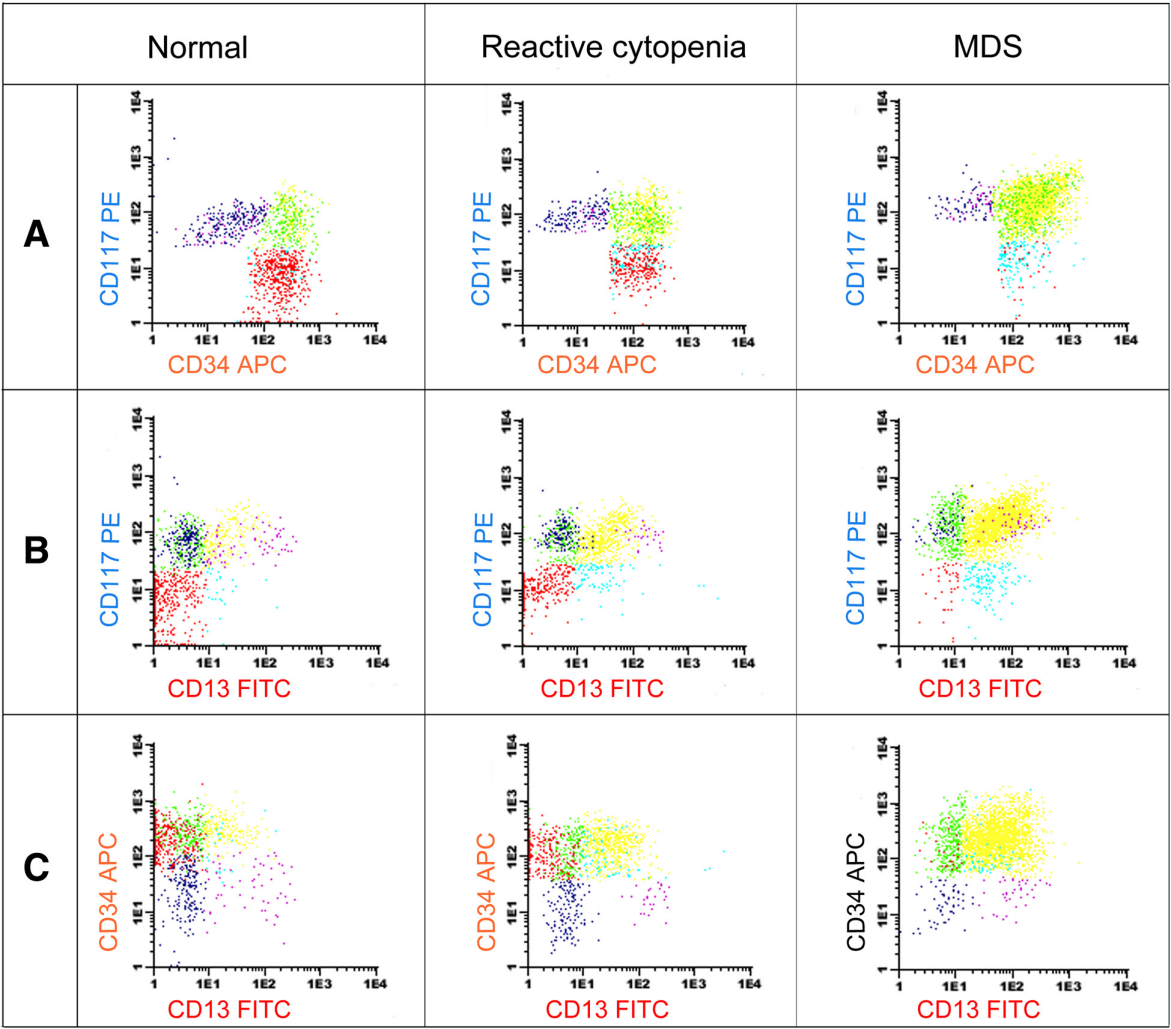
CD34^+^ subsets in normal, reactive cases (idiopathic thrombocytopenia) and MDS (RCMD) analyzed in the CD13/CD34/CD45/CD117 combination. Red: CD34^+^/CD117^−^/CD13^−^ cells that represent the immature precursors and B-cell precursors. Cyan: CD34^+^/CD117^−−^/CD13^+^ cells characterizing the immature myeloid precursors. Green: CD34^+^/CD117^+^/CD13^−−^ representing early myeloblasts and early pro-eritroblasts. Yellow: CD34^+^/CD117^+^/CD13^+^ cells (myeloblasts). Purple: CD34^−−^/CD117^+^/CD13^+^ cells (promyelocytes). Blue: CD34^−^/CD117^+^/CD13^−−^ cells = proeritroblasts. The maturation patterns can be analyzed in the CD34/CD117 combination **(A)**, CD13/CD117 combination **(B)** and CD13/CD34 combination **(C)**. The myeloblasts (yellow) are increased in MDS.

CD34^+^ cells were separated in the SSC/CD34 dot plot [10,19] (Figure 1) and their co-expression of CD19, CD10, CD13, CD117, CD7 and CD56 was examined. The B-cell precursors (CD34^+^/CD19^+^/CD10^+^) were measured as percentage of the total nucleated cells.

All immunophenotypic features were compared with the values found in normal BM. We computed the total number of granulocytic and monocytic abnormalities, as well as those of CD34^+^ cells. We also computed the sum of all abnormalities found.

### Statistical analysis

The mean values and standard deviation were obtained for all variables analyzed. The difference between groups was assessed by analysis of variance. The normal distribution of the features of normal hemopoiesis was examined by the Kolmogorov-Smirnov test, and those with a non-normal distribution were submitted to a log transformation. The relation of B-cell precursors with age and among the groups studied was examined using the Spearman rank order correlation and multiple regressions. Values were considered significant when p < 0.05. The Winstat and SPSS.15 softwares were used for the calculations.

## Results

### Patients’ characteristics

Concerning patients with MDS, the majority were RCMD (Table 1). In 5 cases no mitoses were available for karyotyping. As some of the patients’ groups in IPSS-R and WPSS classification were rather small, we grouped the cases with <5% BM blast (low risk) and MDS with > 5% BM blast (high risk) for analysis. A major part of the patients were low and intermediate risk in all clinical scores analyzed and had BM blasts <5%.

The cases with non-clonal cytopenias included deficiency anemias (n = 11), drug-induced cytopenias (n = 6), aplastic anemia (n = 3), idiopathic thrombocytopenic purpura (n = 4), auto-immune diseases (n = 4), thyroid dysfunction (n = 3), and infection-associated leucopenia (n = 4). There were 15 men and 20 women with a median age of 60 years (14–86).

The normal values were established by the analysis of 41 normal donors of allogeneic bone marrow transplantation with a median age 32 years (15–69); 25 males and 16 females. All features examined except for MFI for SSC in the granulocytic precursors and total B-cell precursors presented a normal distribution. So we used the mean values obtained ± 2 standard deviations except for MFI SSC of myelomonocytic cell lines and percentage of total B-cell precursors. For these two features we used the 5% and 95% percentiles, due to the large variation observed in the normal controls.

### Immunophenotypic analysis

#### Reactive cytopenias

Flow cytometric data of non-clonal cytopenias are shown in Table 2. There was no abnormality concerning SSC of granulocytes and monocytes. Shift to the left, with asynchrony of antigen expression was found in 7 cases (Table 3). In two cases, abnormal maturation pattern in CD13 or CD16 was observed in maturing granulocytes. There was one case with an autoimmune disorder presenting expression of CD34 in maturing granulocytes without any other abnormality, and one with hepatitis C at diagnosis presenting expression of CD7 in maturing granulocytes. In 9 cases (26%), increased percentages of monocytes were found.

**Table 3:** **Frequencies of several abnormalities detected in non-clonal cytopenias and MDS**

**Alterations**	**Reactive cytopenias (n = 35)**	**MDS low risk <5%BM blast (n = 42)**	**MDS high risk >5% BM blast (n = 12)**	**p value for X** ^**2**^
**Granulocytic series**				
Abnormal granularity	0	17	3	**0.001**
Abnormal decrease in CD45 expression	3	9	1	0.245
Abnormal pattern in CD13/CD16	2	23	8	**0.0001**
Abnormal pattern in CD33/HLA-DR	7	26	9	**0.0001**
Expression of CD56	0	9	1	**0.018**
Aberrant expression of CD34	1	7	3	0.06
Aberrant expression of CD7	1	2	1	0.776
Aberrant expression of CD19	0	4	0	0.101
Asynchronous shift to the left	7	11	6	0,227
**Monocytes series**				
Abnormal granularity	7	12	0	**0.10**
Abnormal % Monocytes	11	27	9	0.04
Increase of % Monocytes CD16^+^	8	19	8	0.015
Abnormal pattern in CD33/HLA-DR	5	21	2	**0.01**
Expression of CD56	6	14	3	0.20
Aberrant expression of CD34	1	17	6	**<0.0001**
Aberrant expression of CD7	1	9	5	**0.008**
Aberrant expression of CD19	1	5	2	0.278
**Cells in the blast gate**				
Increase of % CD34^+^ cells	1	11	10	<0.0001
Increase of % CD34^+^/CD13^+^/CD117^+^	4	18	9	0.001
Anomalous expression of CD7	0	9	8	<0.0001
Anomalous expression of CD56	2	15	9	<0.0001

There was an increase of CD16^+^ monocytes in 8 cases of reactive cytopenias. A statistical difference was found among normal, reactive cytopenias and MDS. CD56 was expressed in monocytes in 2 cases, CD7 and CD19 were expressed in one case each (cases with deficiency anemia).

The percentage of total CD34^+^ cells and %CD34^+^/CD117^+^/CD13^+^ cells (myeloid blasts) were increased in only one case of deficiency anemia, associated with a shift to the left in the granulopoiesis. CD56 was expressed in 2 cases (0.06% and 0.09% of the cells). B-cell precursors were below the normal value in 14 cases (41%). All these alterations resulted in an increased number of total abnormalities.

### Abnormalities observed in MDS

The abnormalities found in MDS are also shown in Tables 2 and 3. Most of the aberrancies studied were more common in MDS than in reactive cases. Concerning CD34^+^ cells, they were increased in 26% of the cases of MDS with BM blast count <5% in cytology and in 83% of the cases with RAEB. The same was true for the cells with the phenoptype CD34^+^/CD13^+^/CD117^+^ (Table 3). Abnormal co-expressions were rare in reactive cytopenias and common in MDS (Table 3).

### Relation between B-cell precursors and age

Considering all the subjects studied, the median age was 55 years. Within the groups, normal subjects had a median age of 32 years (range: 15–69); reactive cytopenias: 60 years (range: 14–86) and MDS: 69 years (range: 15–84).

**Figure 2:**
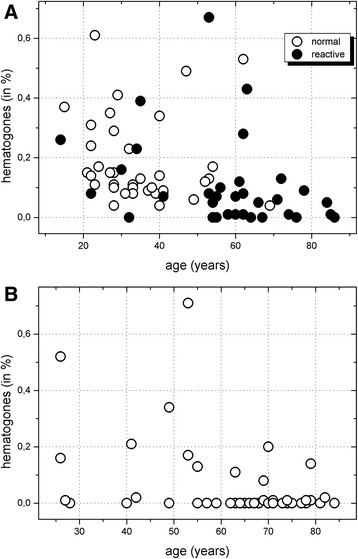
Distribution of bone marrow B-cell precursors according to the age of the patients. A- Variation observed in normal controls and reactive peripheral cytopenias. Decrease is more pronounced in subjects > 55 years old. **B**- **B-cell precursors in MDS.** The number of cells is very low, and frequently absent in patients >55 years old, but it can be present in younger persons.

In the Spearman’s test, there was a negative correlation between age and BCP in all three groups: r = − 0.328; p 0.023 for healthy donors; r = − 0.321; p = 0.032 for reactive cytopenias (Figure 2A) and r = − 0.412; p = 0.002 for MDS cases (Figure 2B). These cells were absent in none of the normal donors, in 8/35 cases of reactive cytopenias and in 33/54 cases of MDS.

Among the subjects with age <55 years, mean B-cell precursors were 0.18%, 0.14% and 0.17% in normal controls, reactive cytopenias and MDS cases respectively, which was not significantly different. Among subjects >55 years old, mean values were 0.28%, 0.075% and 0.015% respectively. These values were significantly different (p < 0.005).

In the multiple regressions considering age, total % CD34^+^ cells, BCP and number of alterations in CD34^+^ cells, the relation could be described in non-clonal subjects (normals and reactive cytopenias) by the equation:

$$ \begin{array}{l}\%\ BCP = \left(-7.3 \times log\  age\right) + \left(8.2 \times log\ \%\ CD{34}^{+} cells\right) + 0.46\\ {}\ \mathrm{corrected}\ {\mathrm{R}}^2 = 0.265\end{array} $$

For the MDS cases the equation was:

$$ \begin{array}{l}\%BCP = \\ {}\left(-7.97 \times log\  age\right) + \left(4.24 \times log\%\ CD{34}^{+} cells\right)\ \hbox{--}\ \left(0.22 \times nr. alterations\ CD{34}^{+} cells\right) + 0.577\\ {}\ \mathrm{corrected}\ {\mathrm{R}}^2 = 0.467\end{array} $$

## Discussion

Several immunophenotypic abnormalities have been described in MDS. According to the WHO 2008 recommendations [1], the finding of three or more abnormalities is considered highly suggestive of MDS. Thus, immunophenotyping has been considered a useful adjuvant method for the diagnosis of MDS in cases with low BM blast counts and a normal karyotype [2]. This technique is also widely used for the diagnosis and assessment of minimal residual disease in several other hematological neoplasms [35,36].

Knowledge of normal antigen expression of the several hemopoietic cells and its changes during normal cell maturation is essential to assess abnormalities and to define leukemia-associated immunophenotypes (LAIPs) [3,8]. It is also advisable for each laboratory to establish their own reference normal values for each panel of monoclonal antibodies used. In healthy individuals, normal maturation of BM precursors is genetically tightly controlled, leading to predictable patterns of antigen expression at different stages of cell maturation. Neoplastic cells are characterized by a deviation of this pattern, as well as by the presence of aberrant cross-lineage antigen expressions [4,5,8,12,37].

In previous studies [10,12,19,23], we have shown that a rather small four-color panel of monoclonal antibodies analyzing the myelomonocytic lineage and the subsets of CD34^+^ cells was suitable to confirm the diagnosis of MDS and allowed us to detect independent prognostic features [23]. Among the phenotypic abnormalities found, those concerning CD34^+^ cells were the most important to predict a shorter survival of the patients. At last we substituted the combination for analysis of dendritic cells and basophils for a combination (CD7/CD56/CD45/CD34) to assess the most frequent leukemia-associated phenotypes. Both minor populations are altered in MDS, but are less important for the differential diagnosis of clonal and non-clonal cytopenias.

In the present work, our aim was to examine which variables detected by our panel were more important to discriminate between reactive PB cytopenias and MDS, especially in those cases with few BM blasts and a normal karyotype. Concerning the variables analyzed, our reactive cases never presented a decreased SSC or a maturation block in the myeloid series. Shift to the left was seen in 7 cases and abnormal expression of CD13 was seen in 2 cases. This is in keeping with the fact that, although variation in antigen expression may occur in reactive cytopenias, the rupture of the normal pattern of maturation should never be seen, as this is indicative of a clonal disorder.

Concerning monocytes, increase in number and expression of CD16 and CD56 that are indicative of cell activation, were observed, although this was more frequent in MDS, as it also has been observed by others [31].

The main immunophenotypic features distinguishing low-risk MDS from reactive cytopenias were the increase of CD34^+^ cells, especially in the presence of normal blast counts in cytology, increase in CD34^+^/CD117^+^/CD13^+^ cells, decrease in B-cell precursors, and aberrant co-expressions in CD34^+^ cells (CD7 or CD56 or decrease in CD13). The presence of anomalous expression of CD7 in CD34^+^ cells was more frequent in high-risk MDS and might reflect progression to leukemia.

The total number of phenotypic abnormalities was also significantly higher in clonal disorders, confirming previous results of our group and others [12,19,24-28]. Overall, in patients with reactive cytopenias, if phenotypic abnormalities are found, a close follow-up of the patient should be made in order to detect a possible evolution to an overt MDS.

The decrease of B-cell precursors has been considered a hallmark of MDS [6,9,13,21,31], even in children. This variable has also been included in phenotypic scores for diagnosis of MDS, such as that proposed by Ogata and included in the guidelines of ELN [31]. B-cell precursors may be assessed as CD34^+^ cells with a low SSC or by their phenotype, which is the way to obtain more reliable results. We also assessed these cells as their percentage among all cells examined and not as percentage of all CD34^+^ cells, as has been recommended by several authors [11,24,30,37]. In MDS it is expected that myeloid progenitors may be increased, provoking a false relative decrease of B-lymphoid precursors. So, the best way to evaluate their number would be to use their percentage among all cells.

On the other hand, it is well known that the number of BM B-cell precursors have a strong variation with age [38]. This was also the case in the present study. We could show that in a multiple regression, their number was dependent of age and the total number of CD34^+^ cells. In MDS, also the total alterations observed in CD34^+^ progenitors entered the equation.

In subjects with age below 55 years the difference in number of BCP was not so pronounced, but in older patients, their number was below normal in reactive cytopenias and this was more pronounced in MDS. This is in keeping with an ageing process of the immune system, which is highly variable with age and amount of exposure to antigen stimulation. The pathophysiology of the decrease of B-cell precursors observed in MDS is not well understood. But, it has been described that these cells may also present abnormalities in antigen expression in MDS [21] that are more pronounced in cases with a higher number of BM blasts. This could be due to a more pronounced dysfunction of hemopoietic progenitors that loose their capacity to produce the B-cell line.

## Conclusion

In conclusion, an antibody panel focused on the analysis of the myelomonocytic cell line and CD34^+^ cells was satisfactory for the differential diagnosis between reactive PB cytopenias and MDS with low BM blast counts and a normal karyotype. The most specific alterations were found in CD34^+^ cells. The number of BCP was more discriminative in older patients. For young patients it is necessary to compare their number with normal age-matched subjects.

## Abbreviations

MDS: Myelodysplastic syndromes
BCP: B-cell precursors
BM: Bone marrow
PB: Peripheral blood
ELN: European Leukemia Net Working Group
WHO: World Health Organization
IPSS: International Prognostic Scoring System
IPSS-R: Revised International Prognostic Scoring System
WPSS: WHO classification-based Prognostic Scoring System
MoAbs: Monoclonal antibodies
FITC: Fluorescein isothiocyanate
PE: Phycoerythrin
PerCP: Peridin clorophyll protein
APC: allophyicocyanin
BD: Becton Dickinson
SSC: Side-scatter
MFI: Median fluorescence intensity
RA: Refractory anemia
RCMD: Refractory cytopenia with multilineage dysplasia
RAEB: Refractory anemia with excess blasts
ANC: Absolute neutrophil count
LAIPs: Leukemia-associated immunophenotypes
